# AMP kinase activation and glut4 translocation in isolated cardiomyocytes

**Published:** 2010-04

**Authors:** Ingrid Webster, Sven O Friedrich, Amanda Lochner, Barbara Huisamen

**Affiliations:** Department of Biomedical Sciences, Division of Medical Physiology, Faculty of Health Sciences, University of Stellenbosch, Tygerberg, South Africa; Department of Biomedical Sciences, Division of Medical Physiology, Faculty of Health Sciences, University of Stellenbosch, Tygerberg, South Africa; Department of Biomedical Sciences, Division of Medical Physiology, Faculty of Health Sciences, University of Stellenbosch, Tygerberg, South Africa; MRC Cape Heart Centre, Tygerberg, South Africa; Department of Biomedical Sciences, Division of Medical Physiology, Faculty of Health Sciences, University of Stellenbosch, Tygerberg, South Africa; MRC Cape Heart Centre, Tygerberg, South Africa

**Keywords:** AMPK, AICAR, GLUT4, GLUT4 exofacial loop, cardiomyocytes

## Abstract

**Summary:**

Activation of AMP-activated protein kinase (AMPK) results in glucose transporter 4 (GLUT4) translocation from the cytosol to the cell membrane, and glucose uptake in the skeletal muscles. This increased activation of AMPK can be stimulated by a pharmacological agent, AICAR (5’-aminoimidazole-4-carboxamide ribonucleoside), which is converted intracellularly into ZMP (5’-aminoimidazole-4-carboxamideribonucleosidephosphate), an AMP analogue. We utilised AICAR and ZMP to study GLUT4 translocation and glucose uptake in isolated cardiomyocytes.

Adult ventricular cardiomyocytes were treated with AICAR or ZMP, and glucose uptake was measured via [^3^H]-2-deoxyglucose accumulation. PKB/Akt, AMPK and acetyl-CoA-carboxylase phosphorylation and GLUT4 translocation were detected by Western blotting or flow cytometry.

AICAR and ZMP promoted AMPK phosphorylation. Neither drug increased glucose uptake but on the contrary, inhibited basal glucose uptake, although GLUT4 translocation from the cytosol to the membrane occurred. Using flow cytometry to detect the exofacial loop of the GLUT4 protein, we showed ineffective insertion in the membrane under these conditions. Supplementing with nitric oxide improved insertion in the membrane but not glucose uptake.

We concluded that activation of AMPK via AICAR or ZMP was not sufficient to induce GLUT4-mediated glucose uptake in isolated cardiomyocytes. Nitric oxide plays a role in proper insertion of the protein in the membrane but not in glucose uptake.

## Summary

AMP-activated protein kinase (AMPK) plays a key role in maintaining energy homeostasis in the cell^1^ by recognising ATP depletion,^2^ initiating changes to restore cellular ATP levels and inhibiting ATP-utilising anabolic pathways.^3^ In heart and skeletal muscle, AMPK plays an important role in accelerating fatty acid oxidation, FAT/CD36 translocation and fatty acid uptake, as well as glut4 translocation, glucose uptake and glycolysis.^4^,^5^ AMPK is activated by cellular stress, which alters the AMP:ATP ratio – this can be nutrient stress, lack of oxygen or exercise-induced stress.^4^,^6^

The AMPK signalling pathway provides an alternative to the insulin-dependant glucose uptake pathway in muscle. Insulin-stimulated activation of glut4 translocation via activation of phosphatidyl-inositol-3 kinase (PI3-K) and PKB/Akt has been extensively researched. Resistance of muscle to the effects of insulin is a hallmark of pre-diabetes. This reduction in insulin sensitivity is reflected in decreased insulin-stimulated glucose uptake.^7^ Since AMPK activates glucose uptake via a distinctly different signalling pathway than insulin, it may present a mechanism to manipulate pharmacologically to treat this disease. Indeed, in insulin-resistant humans and rodents, regular exercise, known to activate AMPK, enhances insulin sensitivity.^8^-^12^ Repeated activation of AMPK may therefore be a mechanism to improve insulin sensitivity.^13^

Furthermore, it was demonstrated that activation of AMPK is responsible for glucose uptake by hearts subjected to ischaemia, underscoring the importance of this kinase in the pathophysiology of the heart.^14^ AICAR (5’-aminoimidazole-4-carboxamide ribonucleoside) is an adenosine analogue which is taken up into the cells and converted to the monophosphorylated nucleotide, ZMP (5’-aminoimidazole-4-carboxamide-ribonucleosidephosphate), by adenosine kinase.^6^,^15^ It was described as a specific activator of AMPK^16^ in intact cells. In has also been found that ZMP, an analogue of AMP, mimics the effects of AMP on allosteric activation of AMPK,^17^ and the promotion of phosphorylation and activation of AMPK by the AMPK kinase, LKB1^18^ without changing the ATP:ADP or ATP:AMP ratio in the cell.^16^

There are numerous studies showing that AICAR increases AMPK phosphorylation as well as glucose uptake in skeletal muscle, the latter mediated via GLUT4 translocation.^19^-^21^ There are, however, only a few studies to date showing effects of AICAR on glucose uptake in the heart, some utilising papillary muscle,^14^ whereas Jing and Holman used oligomycin to activate AMPK in cardiomyocytes.^22^

GLUT4 protein in the heart is largely confined to an intracellular vesicle storage site in the basal, non-stimulated state.^23^,^24^ It becomes recruited to the cell surface under the influence of insulin^25^-^27^ or stimuli such as contraction or hypoxia. GLUT4 vesicles respond to insulin in a marked and dramatic way, increasing GLUT4 levels in the membrane up to nine times that of basal levels.^28^ The protein is recycled via endocytosis in clathrincoated vesicles.^29^

It has, however, been recognised that translocation of GLUT4 to the membrane, and glucose uptake by the GLUT4 transporters are not always reconcilable. There is a marked discrepancy between glucose uptake and translocation of GLUT4.^30^ Although our understanding of the regulation of GLUT4 translocation from the intracellular to the membrane compartments has expanded rapidly over the past years,^31^ the exact mechanisms governing these events, especially at the site of fusion with the cell membrane, is not fully understood. Events elicited by stimulation with insulin have been the focus of intense research, whereas less is known of those elicited by AMPK activation. However, stimulation with activators of AMPK share some of the downstream signalling events of the insulin pathway.^32^,^33^

We used isolated, adult ventricular myocytes stimulated with AICAR and ZMP to correlate glucose uptake and GLUT4 translocation after AMPK activation. The results demonstrated that in cardiomyocytes, activation of AMPK was not sufficient to affect GLUT4 insertion into the cell membrane and therefore glucose uptake.

## Methods

## Materials

AICAR, ZMP, insulin and Triton X-100 were obtained from Sigma, type 2 collagenase was from Worthington, bovine serum albumin (BSA) fraction V, fatty acid-free from Roche Diagnostics and 2-deoxy-D-[3H]glucose from New England Nuclear. The ECL Western blotting detection reagents, antirabbit Ig, horseradish peroxidase-linked whole secondary antibody were from Amersham Biosciences, UK Ltd, GLUT4 (H-61): sc-7938 rabbit polyclonal antibody was from Santa Cruz Biotechnology Inc., phospho-PKB/Akt (Ser^473^), phospho-AMPK-α (Thr^172^) and Phospho-ACC antibodies were from Cell Signalling technology. The anti-GLUT4 (exofacial loop) was purchased from Chemicon International (Temecula, California, USA), and Zenon Alexa Fluor 488 Rabbit IgG Labeling Kit was acquired from Molecular Probes (Eugene, Oregon, USA). Paraformaldehyde was purchased from Merck (Cape Town, RSA) and Dulbecco’s phosphate-buffered saline (PBS) from Gibco (Grand Island, New York, USA).

## Animals

Male Wistar rats weighing between 250 and 300 g were used in all experiments. Animals were bred and kept in the AAALAC-accredited facility of this institution. The project was approved by the Ethics committee of the Faculty of Health Sciences, University of Stellenbosch and conformed to the *Guide for the Care and Use of Laboratory Animals* of the NIH (Publication No. 85-23, revised 1996). Animals were anaesthetised with sodium pentobarbital (160 mg/kg) before experimentation.

## Preparation of cardiomyocytes

Rod-shaped ventricular cardiomyocytes were obtained by collagenase perfusion, essentially as described previously.^34^ Isolated cells were filtered through a nylon mesh (200 × 200 μm) and gently spun down (3 min, 100 rpm). The resulting pellet was resuspended in HEPES buffer (10 mM HEPES pH 7.4, 6 mM KCl, 1 mM Na_2_HPO_4_, 0.2 mM NaH_2_PO_4_, 1.4 mM MgSO_4_, 128 mM NaCl, 5.5 mM glucose, 2 mM pyruvate, containing 1.25 mM CaCl_2_, 2% BSA (fraction V, fatty acid free) and the cells were allowed to recover for 60 min on a slowly rotating platform. Osmotic fragility coupled to trypan blue exclusion (TBE) and cell morphology were used as indices for assessment of cell viability.^35^ Viable cells varied between 70 and 80% and all cell isolates of less than 70% viability were discarded. At the end of experimentation, the viability of cell preparations was determined with propidium iodide.

## Propidium iodide staining

Propidium iodide (PI) staining was used to assess cell membrane permeability. Nuclear staining by PI was assessed with flow cytometric (FACS) analysis. Cardiac myocytes were incubated with 10 μM PI for 15 min before analysis. Data are expressed as mean fluorescence intensity as a percentage of control. FACS analysis was done with a FACSCalibur using Cellquest 3.3 software (Becton Dickinson, San Jose, California, USA).

## Detection of glucose uptake

Myocyte uptake of 2-deoxy-D-[^3^H] glucose (2DG) was measured as described previously.^34^ Cardiomyocytes (approximately 0.5 mg protein) were placed in a total volume of 750 μl assay medium containing (in mmol/l): KCl 6, Na_2_HPO_4_ 1, NaH_2_PO_4_ 0.2, MgSO_4_ 1.4, NaCl 128, HEPES 10, CaCl_2_ 1.25 plus 2% BSA (fraction V, fatty acid free) pH 7.4, 37°C, equilibrated with oxygen. Cells were equilibrated for 15 min in a shaking water bath (180 strokes/min) with or without phloretin (400 μM) for measurement of non-carrier-mediated glucose uptake. They were stimulated with or without insulin (15 min × 100 nM), AICAR (30 min × 1 mM) or ZMP (30 min × 1 mM) as indicated. Glucose uptake was initiated by the addition of 2-deoxy-D-[^3^H] glucose (1.5 μCi/ml; final 2-deoxy-D-glucose concentration 1.8 μM) and allowed to progress for 30 min before the reaction was stopped with phloretin (final concentration 400 μM). The cells were then centrifuged and the pellet was washed twice with HEPES buffer and dissolved in 1 ml 0.5 N NaOH. An aliquot of this solution was then used to assay protein concentration by the method of Lowry,^36^ and the rest was counted for radioactivity.

## Preparation of lysates for Western blotting

Cardiomyocytes were removed after stimulation with insulin, AICAR or ZMP as described above and placed on ice. The cells were centrifuged and washed three times with ice-cold HEPES buffer without albumin and lysed in 100 μl of lysis buffer (25 mM HEPES, 50 mM β-glycerophosphate, 1 mM EGTA, 1% Triton X-100, 10 mM p-nitrophenyl phosphate, 1 mM Na_3_VO_4_, 2.5 mM MgCl_2_, 10 μg/ml aprotonin, 10 μg/ml leupeptin, 1 mM phenylmethylsulphonyl fluoride and 1 mM 1-4-dithiothreitol, pH 7.4). The lysates were centrifuged at 15 000 × g for 10 min and the supernatants diluted with Laemmli sample buffer for SDS-PAGE. An aliquot of the supernatant was used for protein determination.^37^

## Western blotting

After boiling for five minutes, equal amounts of sample protein from the various fractions were separated on a 10% SDS-PAGE and transferred to Immobilon™-P membranes. Transfer and equal loading were confirmed using the reversible stain, Ponceau red. Non-specific binding sites were blocked with 5% fat-free milk powder in TRIS-buffered saline (TBS) for two hours at room temperature and then incubated with either the phospho-Akt (Ser^473^), GLUT4 (H-61): *sc*-7938 or phospho-AMPK (Thr^172^) primary antibody [1:1000 dilution in TBS plus 0.1% Tween-20 (TBST)] for 16 hours at 4°C. After washing with TBST, the membranes were treated for one hour with 1:4000 dilution of anti-rabbit Ig, horseradish peroxidase-linked whole antibody and the bands were visualised with the ECL method.

## Membrane fractionation for measurement of GLUT4 translocation

In order to determine GLUT4 translocation, cardiomyocytes were fractionated into a sarcolemmal membrane and a cytosolic compartment, essentially as described by Takeuchi *et al*.^38^ After stimulation with the various compounds, the cardiocytes were washed twice with ice-cold HEPES buffer without albumin and then sonicated on ice in TES buffer containing (in mM): Tris-HCl 20, pH 7.5, EDTA 2, EGTA 0.5, sucrose 330 PMSF 1 and 25 μg/ml leupeptin. The homogenate was centrifuged to remove particulate debris (1000 × g at 4°C for 10 min).

The supernatant was further fractionated by ultracentrifugation for 90 min at 40 000 × g at 4°C (Beckman, Ti50). The supernatant obtained was considered to represent the cytosolic fraction. The pellet was suspended in the aforementioned buffer minus sucrose but containing 1% (vol/vol) Triton X-100 plus 1% SDS and rotated at 4°C for 30 min. Afterwards the suspension was ultracentrifuged at 40 000 × g for one hour at 4°C (Beckman, Ti50), and the supernatant used as the membrane fraction.

Protein concentration was determined using the method of Bradford,37 samples were diluted in Laemmli sample buffer, boiled for 5 min and stored at –20°C. GLUT4 content and distribution were determined by Western blotting and suitable antibodies.

## Detection of GLUT4 exofacial loop

Approximately 100 000 cardiomyocytes per sample were used and washed once with PBS. After that, the cells were stained with 1 μg GLUT4 antibody coupled to Alexa Fluor 488 according to the manufacturer’s instructions, and washed with PBS. A fixing step with 1 ml PBS containing 1% paraformaldehyde (PFA) for 15 min at room temperature followed. Samples were centrifuged at 4 × *g* for 5 min. Flow cytometric analysis was done with a FACSCalibur using Cellquest 3.3 software (Becton Dickinson, San Jose, California, USA). A forward-scatter against side-scatter plot was used to distinguish the cells from the debris, as described previously.^39^ The fluorescence signal of the labelled antibody bound to GLUT4 was detected in the FL-1 channel using logarithmic amplification. Positive cells were defined by a fixed gate and expressed as a percentage of the total cell population.

## Statistical analyses

Results are presented as mean ± SEM. The significance of the differences between groups was analysed with either a one-way or two-way ANOVA followed by a Bonferroni correction or with the Students *t*-test where applicable, using GraphPad Prism 5; *p* < 0.05 was considered as significant.

## Results

## AMPK activation

**Fig. 1.**
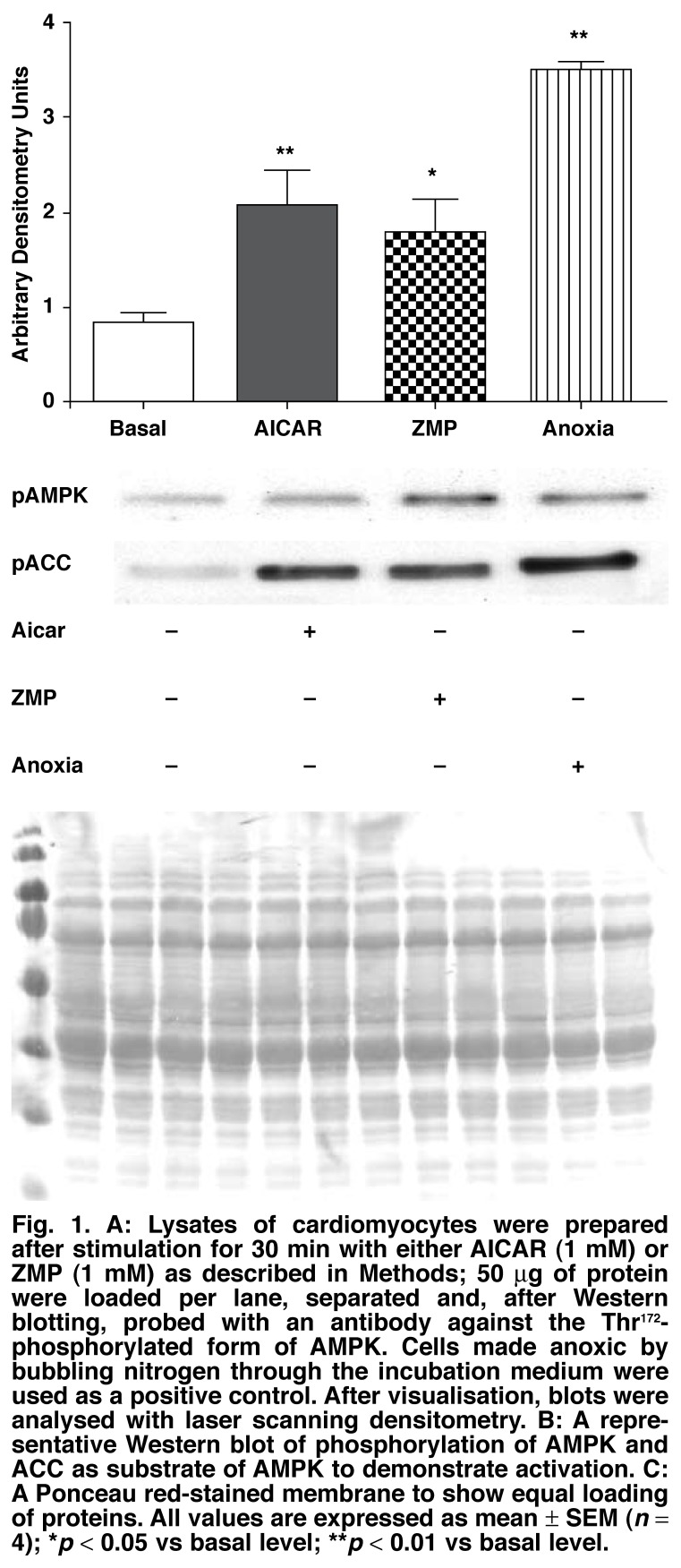
A: Lysates of cardiomyocytes were prepared after stimulation for 30 min with either AI CAR (1 mM) or ZMP (1 mM) as described in Methods; 50 μg of protein were loaded per lane, separated and, after Western blotting, probed with an antibody against the Thr^172^-phosphorylated form of AMPK. Cells made anoxic by bubbling nitrogen through the incubation medium were used as a positive control. After visualisation, blots were analysed with laser scanning densitometry. B: A representative Western blot of phosphorylation of AMPK and ACC as substrate of AMPK to demonstrate activation. C: A Ponceau red-stained membrane to show equal loading of proteins. All values are expressed as mean ± SEM (*n* = 4); *p < 0.05 vs basal level; **p < 0.01 vs basal level.

To determine whether the two pharmacological substances AICAR and ZMP could exert physiological effects via activation of AMPK, the ability of these compounds to elicit phosphorylation of the kinase was determined. This was accomplished by measuring AMPK activation in terms of its phosphorylation on Thr^172^ via Western blotting and a specific antibody. In cardiomyocytes treated with AICAR (1 mM for 30 min) and ZMP (1 mM for 30 min) it was found that both substances resulted in significant phosphorylation of AMPK (Fig. 1A). Cells made anoxic by incubation in medium equilibrated with nitrogen were used as a positive control. To ascertain that the phosphorylated kinase was active, the phosphorylation of one of its downstream substrate proteins, acetyl-Co-A carboxylase (ACC) was determined on the same samples. Fig. 1B is a representative blot showing phosphorylation of AMPK and ACC after stimulation with both AICAR and ZMP, while Fig. 1C is a representative Ponceau red-stained membrane showing equal loading of protein.

## Glucose uptake

**Fig. 2.**
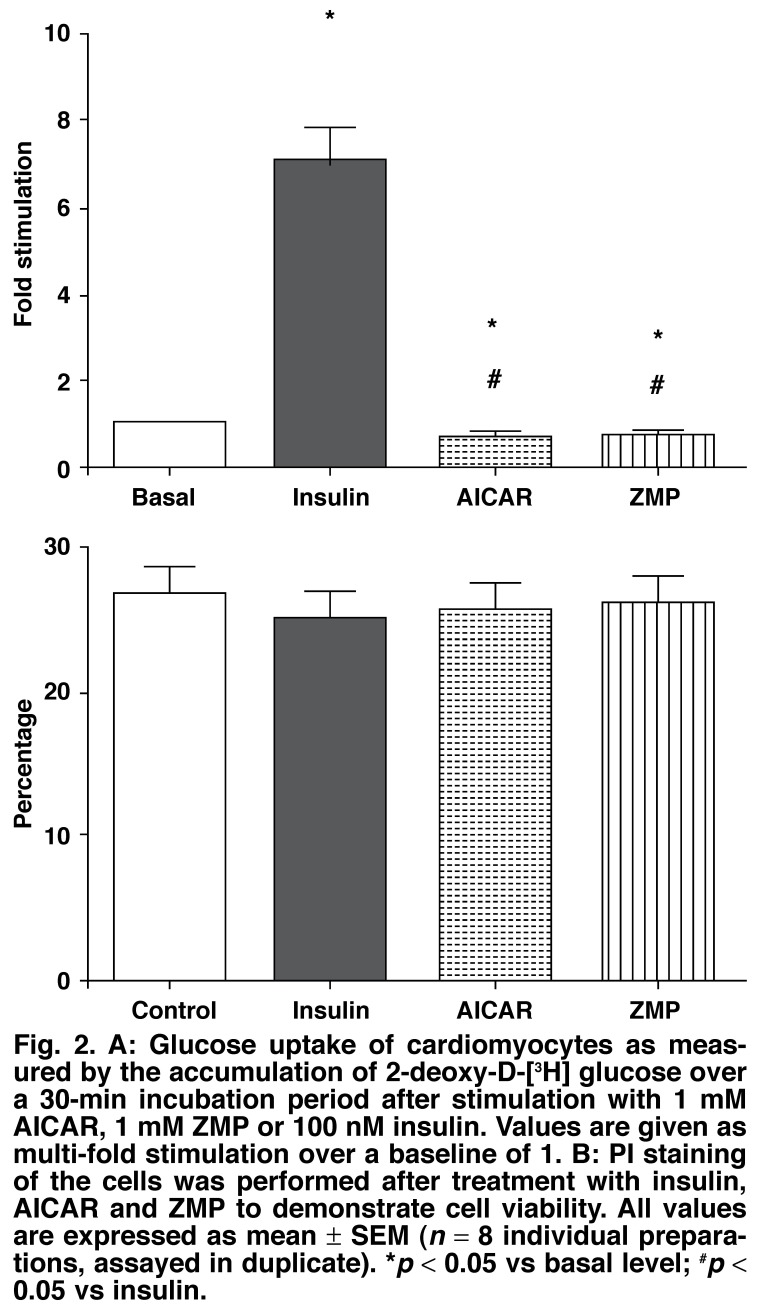
A: Glucose uptake of cardiomyocytes as measured by the accumulation of 2-deoxy-D-[^3^H] glucose over a 30-min incubation period after stimulation with 1 mM AI CAR , 1 mM ZMP or 100 nM insulin. Values are given as multi-fold stimulation over a baseline of 1. B: PI staining of the cells was performed after treatment with insulin, AICAR and ZMP to demonstrate cell viability. All values are expressed as mean ± SEM (*n* = 8 individual preparations, assayed in duplicate). *p < 0.05 vs basal level; ^#^*p* < 0.05 vs insulin.

Insulin (100 nM) increased glucose uptake significantly [7.0 ± 0.71-fold (*p* < 0.05)] from basal levels in the cardiomyocytes, whereas AICAR (1 mM) diminished glucose uptake 0.6 ± 0.1-fold (*p* < 0.05) from basal levels (Fig. 2A).

Javaux *et al*.^40^ reported in 1995 that rabbit cardiomyocytes were unable to phosphorylate AICAR to ZMP. Because of the observed inability of AICAR to elicit glucose uptake in rat cardiomyocytes, we tested the ability of ZMP to affect glucose uptake. As seen in Fig. 2A, ZMP also significantly lowered basal glucose uptake levels 0.6 ± 0.22-fold (*p* < 0.05). To ascertain that this was not because AICAR or ZMP influenced cell viability, PI staining was performed at the end of the experimental protocol. Fig. 2B shows that cell viability was not affected by either substance.

## GLUT4 translocation in cardiomyocytes

**Fig. 3.**
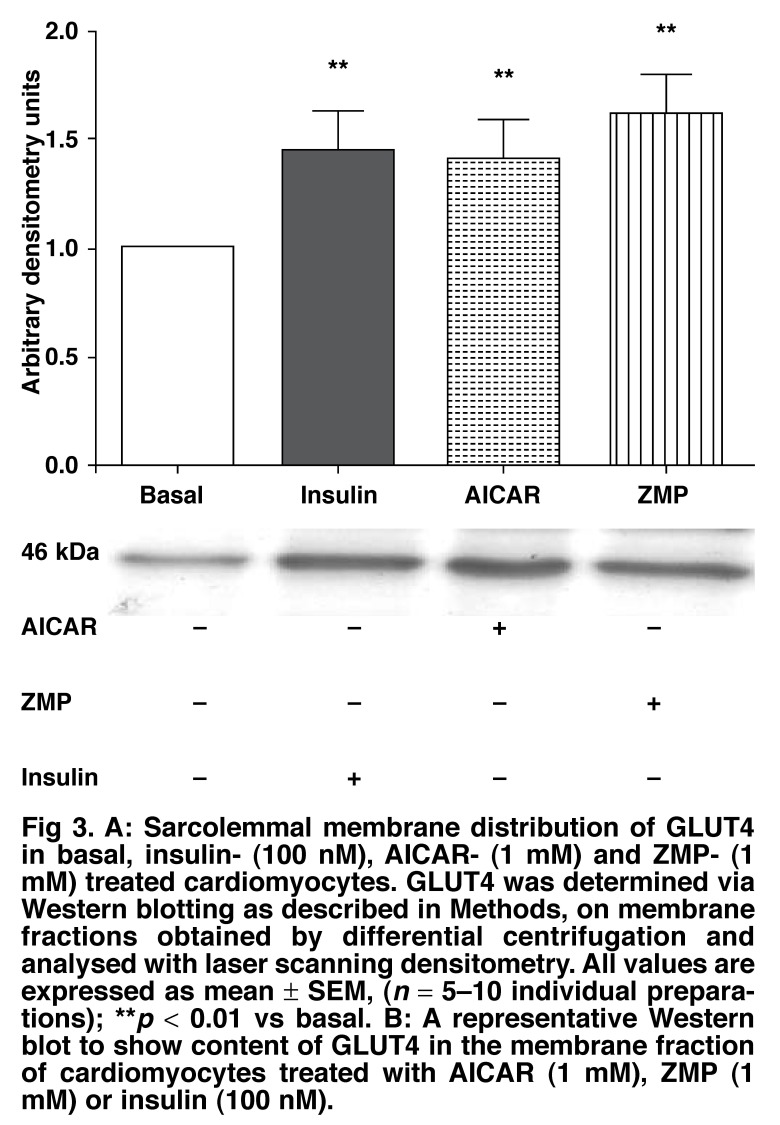
A: Sarcolemmal membrane distribution of GLUT4 in basal, insulin- (100 nM), AI CAR - (1 mM) and ZMP- (1 mM) treated cardiomyocytes. GLUT4 was determined via Western blotting as described in Methods, on membrane fractions obtained by differential centrifugation and analysed with laser scanning densitometry. All values are expressed as mean ± SEM, (*n* = 5–10 individual preparations); **p < 0.01 vs basal. B: A representative Western blot to show content of GLUT 4 in the membrane fraction of cardiomyocytes treated with AI CAR (1 mM), ZMP (1 mM) or insulin (100 nM).

Because of the observed activation of AMPK by AICAR and ZMP, but not of glucose uptake, we assessed GLUT4 translocation under these conditions, fractionating the cells into cytosolic and membrane compartments, and then probing Western blots of the separated proteins with a specific GLUT4 antibody. As shown in Fig. 3A and B, a significant increase in GLUT4 could be seen between basal and stimulated cells in the membrane compartment (1.01 ± 0.01 arbitrary densitometry units vs insulin 1.45 ± 0.2, AICAR 1.29 ± 0.10 and ZMP 1.56 ± 0.1).

## Determination of GLUT4 exofacial loop

**Fig. 4.**
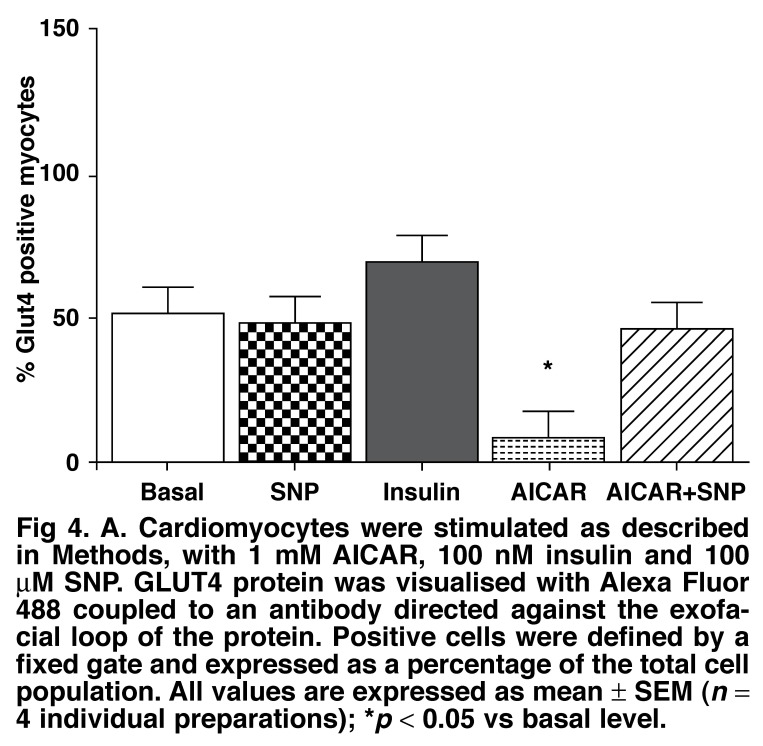
A. Cardiomyocytes were stimulated as described in Methods, with 1 mM AI CAR , 100 nM insulin and 100 μM SNP. GLUT4 protein was visualised with Alexa Fluor 488 coupled to an antibody directed against the exofacial loop of the protein. Positive cells were defined by a fixed gate and expressed as a percentage of the total cell population. All values are expressed as mean ± SEM (*n* = 4 individual preparations); *p < 0.05 vs basal level.

In order to understand this discrepancy, we used an antibody directed against the exofacial loop of the GLUT4 protein, coupled to flow cytometry, to determine whether the protein was properly inserted into the membrane.^41^ As seen in Fig. 4, these results clearly demonstrate that AICAR stimulation of GLUT4 translocation resulted in a protein not exposed on the outside of the cell. In addition, it was demonstrated that insulin treatment led to exposure of GLUT4 on the outside of the cardiomyocyte while AICAR treatment, in accordance with the diminished glucose uptake seen in Fig. 2A, attenuated the amount of protein that could be recognised by the antibody on the outer surface of the cell.

It is postulated that activation of PI-3-kinase or PKB/Akt plays an important role in the docking and fusion of GLUT4 vesicles in insulin-stimulated glucose uptake.^31^,^42^ However, neither AICAR nor ZMP resulted in phosphorylation of PKB/Akt (results not shown).

**Fig. 5.**
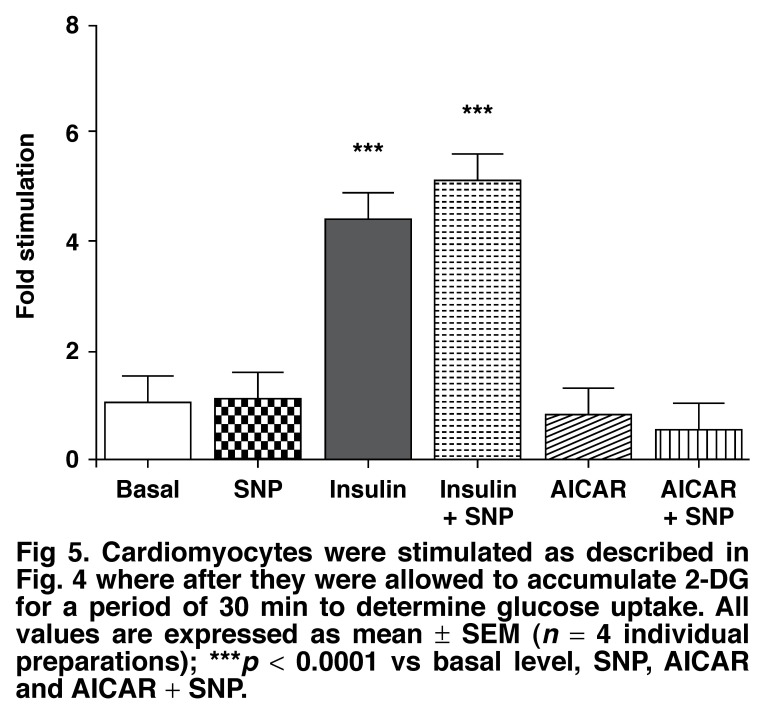
Cardiomyocytes were stimulated as described in Fig. 4 where after they were allowed to accumulate 2-DG for a period of 30 min to determine glucose uptake. All values are expressed as mean ± SEM (*n* = 4 individual preparations); ***p < 0.0001 vs basal level, SN P, AI CAR and AICAR + SNP.

It has also been described that nitric oxide (NO) is important in AMPK-mediated glucose uptake and GLUT4 translocation.^43^ Because myocytes produce much less NO than endothelial cells,^44^ we tested the effects of an NO donor, sodium nitroprusside (SNP) in combination with AICAR on the exposure of GLUT4 on the outside of cardiomyocytes, using the flow cytometric method. As shown in Fig. 4, SNP (100 μm) had no effect on the number of myocytes with the exofacial loop of GLUT4 exposed on the outside under control conditions. However, giving SNP together with AICAR led to enhanced exposure of GLUT4 on the outer surface of the cell. Contrary to expectation, this was not accompanied by enhanced glucose uptake (Fig. 5).

## Discussion

In this study we aimed to determine whether the pharmacological substance, AICAR, known to activate AMPK in skeletal muscle, also exerted similar effects on AMPK activation, glucose uptake and GLUT4 translocation in isolated, adult ventricular cardiac myocytes. Our results showed significantly increased AMPK phosphorylation of Thr^172^ in these cells after stimulation with AICAR (Fig. 1), corroborating findings in EDL skeletal muscle^45^ and hypothalamic cells.^46^ However, Longnus *et al*.^47^ were unable to detect AMPK activation with AICAR in ventricular tissue. In view of the conclusion of Javaux *et al*.^40^ that in cardiomyocytes, AICAR is probably not phosphorylated to ZMP, we similarly tested the effect of ZMP and found increased phosphorylation of Thr^172^ also by this substance. Therefore, both AICAR and ZMP can increase phosphorylation of AMPK in isolated cardiomyocytes.

An increase in AMPK activity leads to stimulation of glucose uptake in skeletal muscle.^48^,^49^ However, the significant AMPK phosphorylation noted in our study was not accompanied by a concomitant increase in cardiomyocyte glucose uptake (Fig. 2A). On the contrary, there was a significant decrease in glucose uptake seen in both the AICAR- and ZMP-treated cells. This finding underscores the work by Jessen *et al*.^50^ which showed that basal glucose transport in AICAR-exposed animals was significantly lower in all muscles when compared to controls or exercised animals. Additionally, Al-Khalili *et al*.^51^ found that both chronic and short-term exposure to AICAR induced AMPK activation in primary human skeletal myocytes but no subsequent increase in glucose uptake.

In contrast to the above, Russell *et al*.,^14^ using a slightly longer incubation time, showed that in heart papillary muscle, incubations with AICAR increased glucose uptake almost twofold and led to AMPK phosphorylation and GLUT4 translocation. Papillary muscle, of course, also contains endothelial and endocardial cells.

Both insulin^25^,^34^ and AMPK^52^,^54^ stimulate glucose uptake by translocation of GLUT4 to the cell membrane. In view of the findings of Russell and co-workers,^14^ we quantified GLUT4 movement to the cell membrane after various stimuli. Fractionating cells into cytosol and sarcolemmal membranes, insulin, AICAR and ZMP treatment resulted in significantly more GLUT4 associated with the cell membrane (Fig. 3). Therefore neither AICAR nor ZMP could stimulate glucose uptake in isolated cardiomyocytes, whereas both substances were able to phosphorylate AMPK and elicit translocation of the GLUT4 transporter from the cytosol to the cell membrane.

The concept that GLUT4 translocation and activation to transport glucose are two independent although interrelated occurrences that can be separated from one another has been put forward by Furtado *et al*.^30^ To substantiate this statement, it was demonstrated that intracellular delivery of PIP3 results in GLUT4 translocation and incorporation into the membrane, while no associated glucose uptake was detected.^57^ In addition, Funaki and co-workers created a cell-permeable phosphoinositide-binding peptide that could induce GLUT4 translocation to the plasma membrane in adipocytes without increasing glucose uptake.^58^

We used an antibody directed against the exofacial loop of the GLUT4 protein, coupled to a flow cytometric method to determine the amount of GLUT4 exposed on the outer surface of cardiocytes after stimulation with either insulin or AICAR. This method clearly demonstrated enhanced exposure with insulin but attenuated exposure with AICAR, similar to the profile of glucose uptake (Fig. 4). As this was in contrast to the results obtained by Davey *et al*.^59^ showing that ischaemia causes a marked translocation of GLUT 4 to the sarcolemmal membrane in whole beating hearts, we speculated on the possible role of NO in this process.

AMPK phosphorylates endothelial nitric oxide synthase (eNOS) on Ser^1177^.^60^ According to Li *et al*.,^61^ this activation modulates glucose uptake and GLUT4 translocation in heart muscle. Although cardiomyocytes also contain NOS, isolated cardiocytes produce very little NO in comparison to cardiac endothelial cells.^44^ We therefore argued that the lack of NO formation and subsequent activation of the cGMP pathway may be responsible for the lack of glucose uptake in our cells. To test this, we supplemented the cells with NO with the addition of SNP. This demonstrated that indeed, simultaneous stimulation with SNP and AICAR resulted in significantly more GLUT4 protein now detectable with the antibody against the exofacial loop of the protein. However, contrary to our expectations, glucose uptake was not affected, underscoring the concept that there still has to be activation of the GLUT4 transporter after insertion into the membrane. These results then reinforce the finding of Li *et al*.^61^ that more signals than NO in addition to AMPK are necessary to induce glucose uptake.

The results obtained in this study therefore argue that other, hitherto unidentified factors besides AMPK activation and GLUT4 translocation are necessary to induce glucose transport via this pathway. We furthermore concluded that these signals were not activated in the isolated cardiomyocytes via activation of AMPK, although they were active in beating cardiac muscle. Both AICAR and ZMP have proved valuable tools in this study.
